# Immunity and Growth Plasticity of Asian Short-Toed Lark Nestlings in Response to Changes in Food Conditions: Can It Buffer the Challenge of Climate Change-Induced Trophic Mismatch?

**DOI:** 10.3390/ani13050860

**Published:** 2023-02-27

**Authors:** Guang Lu, Xinjie Zhang, Xinyu Li, Shuping Zhang

**Affiliations:** 1 Key Laboratory of Ecology and Environment in Minority Areas (National Ethnic Affairs Commission), Minzu University of China, Beijing 100081, China; 2 College of Life and Environmental Sciences, Minzu University of China, Beijing 100081, China

**Keywords:** nestling, immunity, growth, trophic mismatch, physiological plasticity

## Abstract

**Simple Summary:**

Climate change-induced trophic mismatch poses a challenge to nestling survival. This paper presents a physiological adaptation of nestlings to poor food conditions. Some indicators related to the immunity (IFN-γ, TNF-α, and IL-1β) and growth (IGF-1, body mass growth rate) of wild Asian short-toed lark nestlings were studied. The relationships between these indicators and food abundance are analyzed. The results indicate that the plasticity of immunity and growth in nestlings may buffer the challenge of trophic mismatch in birds.

**Abstract:**

Passerine nestlings frequently suffer from sub-optimal food conditions due to climate change-induced trophic mismatch between the nestlings and their optimal food resources. The ability of nestlings to buffer this challenge is less well understood. We hypothesized that poor food conditions might induce a higher immune response and lower growth rate of nestlings, and such physiological plasticity is conducive to nestling survival. To test this, we examined how food (grasshopper nymphs) abundance affects the expression of interferon-γ (*IFN-γ*), tumor necrosis factor-α (*TNF-α*), interleukin-1 β (*IL-1β*) genes, plasma IGF-1 levels, body mass, and fledging rates in wild Asian short-toed lark (*Alaudala cheleensis*) nestlings. Linear mixed models revealed that nymph biomass significantly influenced the expression of *IFN-γ*, *TNF-α*, and *IL-1β* genes, and the level of plasma IGF-1. The expressions of *IFN-γ*, *TNF-α*, and *IL-1β* genes were negatively correlated with nymph biomass and plasma IGF-1 level. Plasma IGF-1 level, nestling body mass growth rate, was positively correlated with nymph biomass. Despite a positive correlation between the nestling fledge rate and nymph biomass, more than 60% of nestlings fledged when nymph biomass was at the lowest level. These results suggest that immunity and growth plasticity of nestlings may be an adaptation for birds to buffer the negative effects of trophic mismatch.

## 1. Introduction

In the middle and high latitudes of the Northern Hemisphere, the nestling growth period of passerines must be synchronized with the peak abundance of insect larva prey to maximize nestling growth and survival [1,2,3,4,5]. Climate change-induced spring temperature increases can advance the timing of insect development [6,7], whereas the timing of breeding in birds is regulated more by photoperiod than temperature [8], resulting in a trophic mismatch between nestlings and insect larvae [7,8,9,10,11,12]. Nestlings hatching outside the peak period of insect larvae abundance may suffer malnutrition, posing a significant challenge to nestling survival [9,10]. Although adults increase their feeding efforts to satisfy nestling nutritional requirements [13], little is known about how nestlings cope with the stress caused by trophic mismatch. Whether nestlings that hatch during periods when food is less abundant buffer such nutritional challenges using their own resources remains unknown. Understanding how nestlings cope with trophic mismatch may provide insight into the ability of birds to adapt to climate change.

Phenotypic plasticity is the ability of organisms to change gene expression in response to short-term environmental challenges [14]. Several food-controlled experiments have demonstrated physiological plasticity in nestlings in response to changes in food conditions [15,16,17]. For example, when food is insufficient, House sparrow (*Paaser domesticus*) nestlings can prioritize bone growth, whereas Zebra finch (*Taeniopygia guttata*) nestlings can adjust their growth rate during different developmental stages [15,16]. Therefore, the physiological plasticity in nestlings could be an important mechanism for coping with sub-optimal food conditions. When food is scarce, the limited resources of nestlings must be allocated between growth and survival [18,19,20,21]. The immune system is critical for the survival of animals because it serves as a defense mechanism against pathogenic organisms [22,23]. Pathogen infection experiments indicate that maintaining immune function consumes a significant amount of nutrients and energy, which can inhibit growth [24,25,26,27,28,29]. Other studies suggest that short periods of poor nutrition enhance nestling immunity [30,31,32]. These findings suggest that temporary food shortages may activate immune response while reducing growth to ensure the short-term survival of nestlings.

Immune cytokines and insulin-like growth factor-1 (IGF-1) are important immune and growth factors in birds. Immune cytokines are small soluble protein molecules produced by immune cells such as T lymphocytes and macrophages in response to immunogen or other stimuli [33,34]. Studies in humans have shown that inadequate nutrition can induce macrophages to produce immune cytokines [34,35]. Interleukin (IL), interferon (IFN), and the tumor necrosis factor (TNF) superfamily are the primary cytokines. For example, IL-1 and TNF-α can activate T and B lymphocytes, stimulating the production and release of antibodies [36,37], whereas IFN can stimulate peripheral cells to inhibit virus replication [38]. Insulin-like growth factor 1 (IGF-1) is an important indicator reflecting bird growth [39,40,41,42,43]. For example, plasma IGF-1 level was positively correlated with the nestling growth rate of wild Great tits (*Parus major*) [42]. IGF-1 activates protein synthesis by changing gene transcription levels [41,44] and increases muscle mass by promoting cell growth and differentiation [39,45]. An increase of immune cytokines, especially IL-1, can activate the hypothalamic-pituitary-adrenal axis, leading to the secretion of corticosterone, which has been known to inhibit the activity of IGF-1 [46,47,48,49]. Therefore, immune cytokines inhibit the activity of IGF-1. This physiological pathway could explain why maintaining immunity may slow down growth. 

Individual variation in hatching dates frequently occurs in passerine bird populations [50,51,52,53]. Variation in hatching dates is considered an ecological benefit because it is a way of partitioning niches along the time axis to avoid competition for breeding resources within a population [54]. While most adults tend to synchronize the nestling hatch date with the insect larva peak, some nestlings hatch outside the peak period of insect abundance [55,56]. This natural variation among individuals provides a model for evaluating the ability of nestlings to cope with food challenges. The Asian short-toed lark (*Alaudala cheleensis*) is the dominant passerine of the Hulun-Beir grassland in Inner Mongolia, China. Asian short-toed larks exhibit significant within-population variation in the timing of breeding, as is typical of passerines. Most individuals begin egg-laying in mid-May, while others start later in late April or May [57]. Asian short-toed lark nestlings are predominantly fed grasshopper nymphs (*Orthoptera*) [56]. The proportion of nymphs in the diet of nestlings hatching outside the period of peak grasshopper nymph abundance was significantly lower than that of nestlings hatching within the period of peak grasshopper nymph abundance. The proportion of beetles and grass seeds in nestlings’ diets increased with decreasing nymph abundance. Moreover, the nutrients and energy that nestlings obtained from alternative diets were significantly lower than that from grasshopper nymphs. Plasma glucides, amino acids, tricarboxylic acid (TCA) cycle metabolites, and some fatty acids of the nestlings hatched under medium or low nymph abundance conditions were significantly lower than those of nestlings hatched under high nymph abundance conditions [56]. We found that Asian short-toed larks in Hulun-Beir, Inner Mongolia, are affected by climate change-induced trophic mismatch, where abnormally high or low spring temperatures in some years caused significant asynchrony between peak hatching and peak grasshopper nymph abundance [57]. The peaks of nymph abundance in 2014 and 2016 occurred 12 days earlier and later than in 2015, respectively, which resulted in a complete mismatch between the hatching peak and the nymph peak period in these two years [57]. As a result of this “trophic mismatch”, most nestlings hatched outside the peak period of grasshopper nymph abundance and suffered poor food conditions. Therefore, Asian short-toed larks are a suitable case species for investigating the capacity of nestlings to respond to food challenges.

In this article, using the Asian short-toed lark population as a model, we hypothesize that poor food conditions may induce a higher immune response and lower growth rate of nestlings, and such physiological plasticity is conducive to nestling survival. To test this hypothesis, we examined *IFN-γ*, *TNF-α*, and *IL-1β* expression, plasma IGF-1, body mass, and fledge rate per nest in a field population of Asian short-toed lark nestlings that hatched during different periods of grasshopper nymph abundance and analyzed the relationships between these physiological and ecological indicators.

## 2. Materials and Methods

### 2.1. Study Site

The study area is located in the Hulun Lake National Nature Reserve (47°45′50″ N–49°20′20″ N, 116°50′10″ E–118°10′10″ E) in the northeastern part of the Inner Mongolian Autonomous Region, China. This is a semiarid, steppe region that has long, severe winters and short summers. The mean annual temperature, precipitation, and potential evaporation are −0.6 °C, 283, and 1754 mm, respectively. The dominant plant species are *Stipa krylovii*, *Leymus chinesis,* and *Cleistogenes squarrosa*.

### 2.2. Data Collection

**Figure 1:**
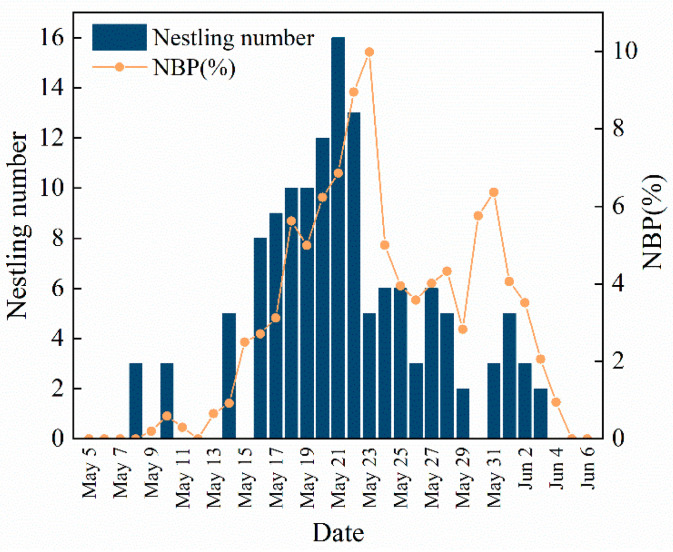
Daily nymph biomass proportion (NBP) and the number of newborn Asian short-toed lark (*Alaudala cheleensis*) nestlings. NBP is the proportion of the daily grasshopper nymph biomass relative to the total biomass measured on all survey days.

We monitored Asian short-toed lark nests in the study area daily from 15 April to 15 June 2019, recording nestling hatching dates, body mass, age, brood size, and the number of nestlings that survived to fledgling. Fledge rate of nestlings is the percentage of a fledgling number relative to the total nestling number hatched in one day, excluding the preyed nestlings. The relative abundance of grasshopper nymphs in the study area was quantified by catching these in an insect net on 10 parallel, 2 m × 100 m sampling transects, spaced 10 m apart, daily. Captured nymphs were dried in a drying oven at 70 °C for 24 h and weighed to determine their biomass. The mean daily nymph biomass was the average daily biomass obtained from all 10 transects. We used “nymph biomass proportion” (NBP), the proportion of the daily nymph biomass relative to the total biomass measured on all survey days, as a measure of daily grasshopper nymph abundance. Totally 135 nestlings were recorded. Asian short-toed lark nestlings can fly 8 days after hatching. We use the NBP on the fourth day after sample nestlings hatched as an indicator of the quality and quantity of food available to that nestling. Daily NBP and the number of newborn nestlings are shown in Figure 1. The daily mean ambient temperature during the experiment varied from 7.54 to 17.39 °C. Because variation in the mean ambient temperature during different periods of the study may also have affected the immune status of nestlings [58], we also measured the nest temperature daily at 6:00 am with a FLIR C2 infrared thermometer when nestlings were four days old.

### 2.3. Nestling Blood Samples

A 100 uL peripheral blood sample was collected from the first hatched four-day-old nestling in each nest at 6:00 am. The brachial wing vein of each nestling was punctured with a disinfected 23 G needle within 1–3 min of capture, and blood exuding from the puncture site was collected into heparinized microcapillary tubes. The skin around the puncture site was disinfected with medical alcohol before and after puncturing. Pressure was applied to the puncture site for 1 min with an alcohol-soaked cotton wool swab to staunch bleeding. Blood samples were centrifuged at 4000 r/min for 20 min to separate the plasma and blood cells. The resultant plasma and blood cells were snap-frozen in liquid nitrogen and stored at −80 °C.

### 2.4. Cytokine Gene Expression Analysis

The total RNA of blood cell samples was isolated from 1 mL TRIzol reagent in 200 μL chloroform, centrifuged at 12,000 r/min for 5 min at 4 °C, after which about 200 μL of the supernatant was transferred to a clean RNase-free centrifuge tube. 200 μL chloroform was then added to the tube, which was centrifuged at 12,000 r/min for 15 min at 4 °C. 150 μL of the supernatant was then transferred to a clean RNase-free centrifuge tube to which 150 μL isopropanol was added and the tube centrifuged at 12,000 r/min for 15 min at 4 °C. The resultant RNA pellet was washed with 400 μL 75% ethanol (dissolved in DEPC water) two times, briefly air dried for 5 min, and dissolved in 30 μL DEPC water. Total RNA quality and quantity were evaluated using agarose gel electrophoresis (AGE) and a NanoDrop 2000 spectrophotometer. After validation, 2 μg of the total RNA sample was reversely transcribed to cDNA using M-MLV Reverse Transcriptase with oligo dT primers. The cDNA was used as a template for RT-qPCR. Primers were designed based on *IFN-γ*, *TNF-α*, and *IL-1β* mRNA sequences in NCBI using Primer 5 software (Table 1). The RT-qPCR protocol was as follows; pre-degeneration at 95 °C for 1 min, degeneration at 95 °C for 15 s, annealing at 59 °C for 15 s and extension at 72 °C for 40 s, for a total of 40 cycles. After the reaction, a melting curve analysis from 55 to 95 °C was applied to ensure the consistency and specificity of the amplified product. The GAPDH expression was confirmed stable under all treatments, and this gene was consequently used as the reference gene to normalize mRNA levels among samples. RT-qPCR was performed twice in triplicate. The values of the average cycle threshold (Ct) were determined, and Ct scores for gene transcripts in each sample were normalized using the ΔCt scores for GAPDH and expressed as the fold change in gene expression using the equation 2^−ΔΔCT^.

### 2.5. Plasma IGF-1 Analysis

IGF-1 concentration was measured using enzyme immunoassay kits from Enzo Life Sciences (New York, NY, USA). Bound IGF-1 was separated from the binding protein with 12.5% 2 mol/L HCl and 87.5% ethanol solution according to the methods in Sparkman et al. [59]. The kits have been validated for the Asian short-toed lark by serial plasma dilutions. All serum samples were 1:5 diluted (10 μL of the sample and 40 μL of the sample dilution) and added to sample wells in triplicate. The respective inter- and intra-plate coefficients of variation were 5.2% and 7.7%.

### 2.6. Statistical Analysis

The effects of nymph biomass, nest temperature, and their interaction on the plasma IGF-1 levels, *IFN-γ*, *TNF-α*, and *IL-1β* gene expression levels in the blood cells of nestlings were analyzed with linear mixed models (LMMs). Regression analysis is used to analyze the relationship between nymph biomass and plasma IGF-1, the expression of cytokine genes, body mass, and survival rate per nest. *p*-values < 0.05 were considered significant. The LMM analyses were performed in SPSS version 24 (SPSS Inc., Chicago, IL, USA), and the regression analyses were performed in R (version 3.6.2).

## 3. Results

### 3.1. Blood Cell Cytokine Gene Expression of Nestlings

**Figure 2:**
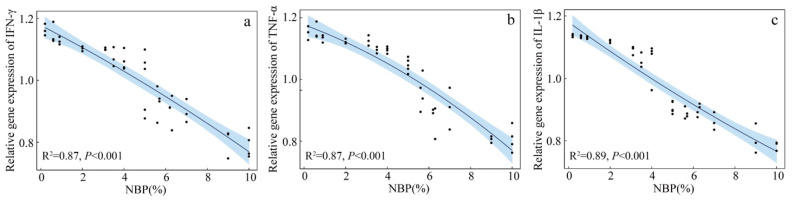
The relationship between grasshopper nymph biomass proportion (NBP) and the relative expression of the interferon-γ (*IFN-γ*) (**a**), tumor necrosis factor-α (*TNF-α*) (**b**), interleukin-1β (*IL-1β*) (**c**) genes of Asian short-toed lark (*Alaudala cheleensis*) nestlings (*n* = 45). NBP is the proportion of the daily grasshopper nymph biomass relative to the total biomass measured on all survey days. The blue area is the confidence interval.

The LMMs of the effects of NBP and nest temperature on *IFN-γ*, *TNF-α*, and *IL-1β* gene expression indicate that only NBP significantly influenced the expression of all three genes (Table 2). Quadratic polynomial regression indicates a significant, negative correlation between NBP and *IFN-γ*, *TNF-α* and *IL-1β* gene expression (*IFN-γ*: R^2^ = 0.87, *p* < 0.001, Figure 2a; *TNF-α*: R^2^ = 0.87, *p* < 0.001, Figure 2b; *IL-1β*: R^2^ = 0.89, *p* < 0.001, Figure 2c).

### 3.2. Plasma IGF-1 Levels and Body Mass of Nestlings

**Figure 3:**
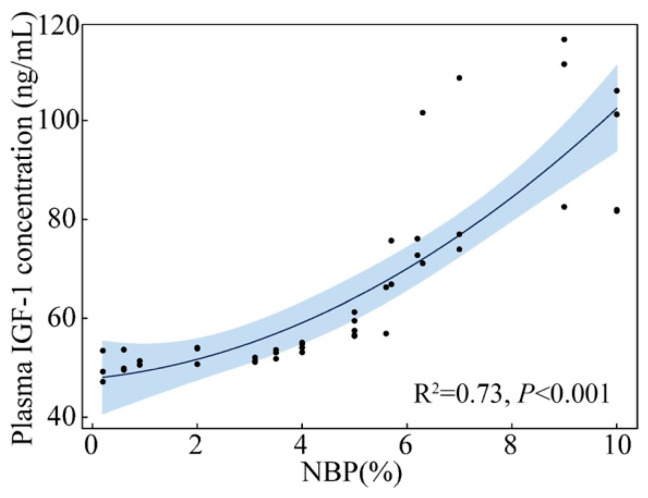
The relationship between nymph biomass (NBP) and plasma insulin-like growth factor-1 (IGF-1) concentrations of Asian short-toed lark (*Alaudala cheleensis*) nestlings (*n* = 45). NBP is the proportion of the daily grasshopper nymph biomass relative to the total biomass measured on all survey days. The blue area is the confidence interval.

**Figure 4:**
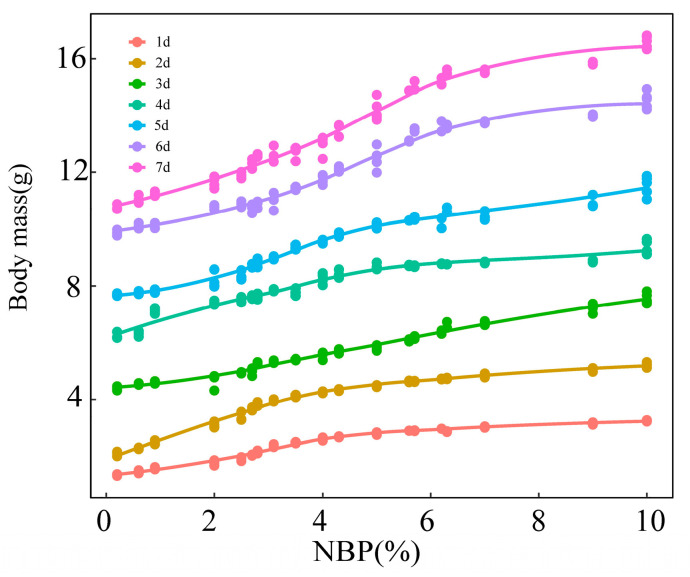
The relationship between nymph biomass proportion (NBP) and body mass of Asian short-toed lark (*Alaudala cheleensis*) nestlings (*n* = 45). 1d, 2d, 3d, 4d, 5d, 6d, and 7d indicates the age of nestlings in days. NBP is the proportion of the daily grasshopper nymph biomass relative to the total biomass measured on all survey days.

**Figure 5:**
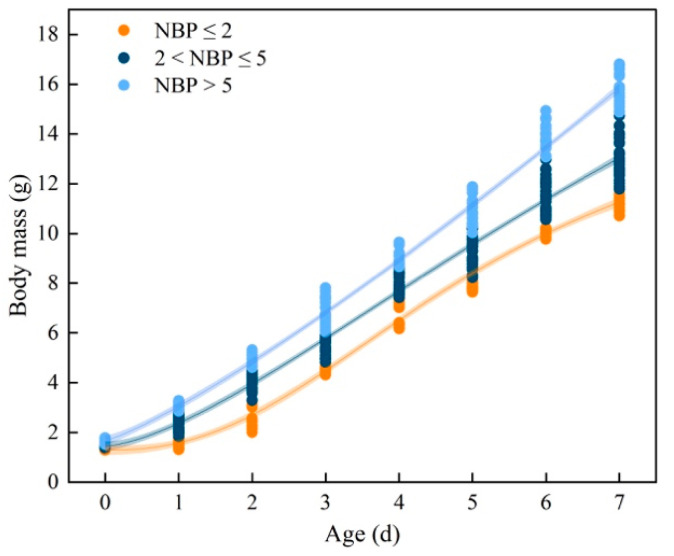
The relationship between body mass and age (days after birth) of Asian short-toed lark (*Alaudala cheleensis*) nestlings (*n* = 45). NBP is the proportion of the daily grasshopper nymph biomass relative to the total biomass measured on all survey days. The shaded area is the confidence interval.

The LMMs of the effects of NBP and nest temperature on nestling plasma IGF-1 concentration indicate that NBP significantly influenced the level of IGF-1 while temperature did not (Table 2). Quadratic polynomial regression indicates a significant positive correlation between NBP and plasma IGF-1 concentration (R^2^ = 0.73, *p* < 0.001; Figure 3). The body mass of one to seven-day-old nestlings increased with NBP increase and approximated a cubic polynomial regression curve (1d: R^2^ = 0.976, *p* < 0.001; 2d: R^2^ = 0.987, *p* < 0.001; 3d: R^2^ = 0.979, *p* < 0.001; 4d: R^2^ = 0.954, *p* < 0.001; 5d: R^2^ = 0.961, *p* < 0.001; 6d: R^2^ = 0.973, *p* < 0.001; 7d: R^2^ = 0.976, *p* < 0.001; Figure 4). We categorized NBP as high (NBP>5%), medium (2% < NBP ≤ 5%), and low (NBP ≤ 2%) based on Figure 1 and assigned nestlings to these groups. The body mass-age relationships of the three groups of nestlings approximated logistic regression curves (NBP ≤ 2%: R^2^ = 0.98, *p* < 0.001; 2% < NBP ≤ 5%: R^2^ = 0.98, *p* < 0.001; NBP > 5%: R^2^ = 0.99, *p* < 0.001; Figure 5). The daily mean slope of the curve (growth rate) was 1.49, 1.72, and 2.11 in the NBP > 5%, 2% < NBP ≤ 5%, and NBP ≤ 2% groups, respectively (Figure 5).

### 3.3. Correlation between Blood Cell Cytokine Gene Expression and Plasma IGF-1 of Nestlings

**Figure 6:**
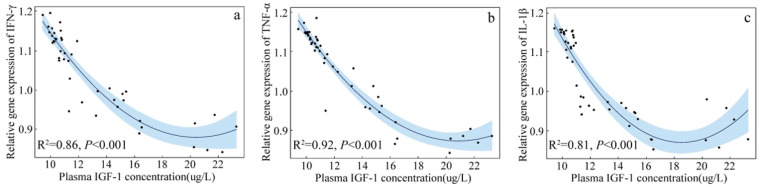
The relationship between plasma IGF-1 concentration and relative expression of the interferon-γ (IFN-γ) (**a**), tumor necrosis factor-α (TNF-α), (**b**) interleukin-1β (IL-1β), (**c**) genes of Asian short-toed lark (*Alaudala cheleensis*) nestlings (*n* = 45). The blue area is the confidence interval.

Quadratic polynomial regression indicates that there was a significant, negative correlation between plasma IGF-1 concentration and blood cell *IFN-γ*, *TNF-α* and *IL-1β* gene expression (*IFN-γ*: R^2^ = 0.86, *p* < 0.001, Figure 6a; *TNF-α*: R^2^ = 0.92, *p* < 0.001, Figure 6b; *IL-1β*: R^2^ = 0.81, *p* < 0.001, Figure 6c).

### 3.4. Fledge Rate of Nestlings

**Figure 7:**
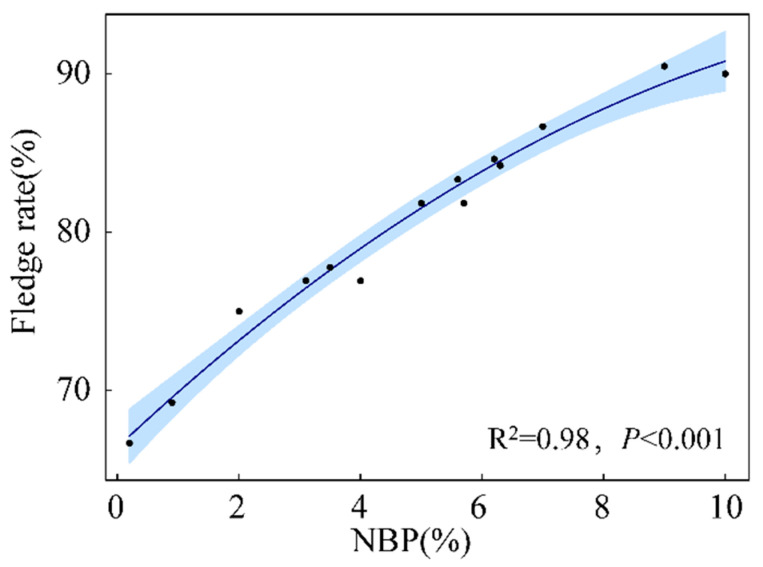
The relationship between nymph biomass proportion (NBP) and fledge rate of Asian short-toed lark (*Alaudala cheleensis*) nestlings (*n* = 45). NBP is the proportion of the daily grasshopper nymph biomass relative to the total biomass measured on all survey days. The blue area is the confidence interval.

Quadratic polynomial regression shows that the fledge rates of nestlings increased with NBP increase (R^2^ = 0.98, *p* < 0.001), and the lowest fledge rate was more than 60% (Figure 7).

## 4. Discussion

The expression of *IFN-γ*, *TNF-α*, and *IL-1 β* genes was negatively correlated with nymph biomass, indicating that the immune systems of nestlings were activated by stimulating immune cells to release cytokines when food conditions were limited. At the same time, there was a significant positive correlation between plasma IGF-1 level and NBP, indicating that the secretion of IGF-1 of nestlings was inhibited when food conditions were limited. The negative correlations between the three kinds of cytokines and IGF-1 indicate that, as found in available studies [46,47,48,49], a higher immune response may inhibit the secretion of IGF-1 in nestlings. These results confirm that poor food conditions can induce a higher immune response, which may negatively influence the growth of nestlings. 

Some available studies in humans have shown that malnutrition can stimulate macrophages to produce cytokines [34,35]. When the energy intake is low, glycolysis, the first stage of the glucose decomposition process, is enhanced to meet energy demands. Studies have shown that increasing glycolytic enzymes, such as pyruvate kinase 2 and glyceraldehyde-3-phosphate dehydrogenase, can induce macrophages to increase the release of IL-1 and TNF-α [60,61]. Therefore, poor nutrition may increase the expression of immune cytokine genes in nestlings by increasing glycolysis. In addition, the secretion of IGF-1 is also closely related to the energy level of the cells; low energy levels can inhibit IGF-1 secretion by activating the AMPK pathway [62]. In agreement with our results, some experimental studies have also shown that nestlings can maintain immunity and reduce tissue growth during short-term food restriction. The authors interpreted these observations as showing that the energy required for maintaining normal immunity is minor compared with the energy required for growth [63,64]. Unlike previous studies, our study tested the gene expression of immune cytokines, signaling molecules that activate the immune system [36,37]. The gene expression of *IFN-γ*, *TNF-α*, and *IL-1 β* increased in nestlings under poor food conditions, indicating that more nestling energy was used in the immune response rather than the immune system needing less energy. Therefore, the results of immune cytokines and IGF-1 levels showed that the energy required for the growth of nestlings was insufficient in poor food conditions, and the energy required for maintaining immune function might be given priority.

The relationship between the body mass of nestlings and NBP and the growth rate difference among different NBP groups implies that the lower IGF-1 level retards the growth of nestlings. In contrast, the results show that nestling survival rates positively correlated with NBP, indicating that poor food conditions cause higher nestling mortality. Nonetheless, the fact that up to 60% of nestlings could fledge even when NBP was at its lowest indicates that most of the nestlings in poor food conditions can survive despite a relatively light body mass. These results, together with the cytokine results, suggest that poor food conditions induced higher immune response might ensure most of the nestlings survive in poor food conditions despite a lighter body mass, thus partially buffering the effect of trophic mismatch. However, such physiological plasticity may only work in the short term because prolonged food shortage caused by severe trophic mismatch will lead to a decline in immune function due to a lack of protein, energy, and other essential nutrients [16,65]. In other words, the physiological plasticity of nestlings appears insufficient to buffer the severe food shortage induced by trophic mismatch. Under the climate change scenario, extreme inclement weather occurs frequently and is seasonally heterogeneous [66,67]. For example, a long-term study found that a cold spring could seriously reduce the abundance of insect larvae, reducing the survival rate of a migrating bird species by more than 50% [67]. According to our previous study, the Asian short-toed larks in our study area have experienced severe trophic mismatch induced by warm and cold springs in 2014 and 2016, in which the peak of nymph abundance was 6 days earlier and later than the peak hatchings in 2014 and 2016, respectively [57]. Although the mismatch days are the same between the two years, the nestling survival rate in 2016 is lower than in 2014. These facts suggest that extreme weather may lead to a more severe food shortage for the nestlings, which may not be mitigated by the physiological plasticity of nestlings.

Inter-sibling competition is a possible factor affecting the survival of nestlings in poor food conditions. Limited food causes increased inter-sibling competition manifesting in increased begging behavior, which means that individuals in the same nest may receive different amounts of food and consequently differ in nutritional status [68,69,70,71]. The nestlings with weak competitiveness may be unable to obtain essential energy and nutrients to survive. Therefore, although the plasticity of immunity and growth may maintain the short-term survival of some nestlings, it cannot guarantee the survival of severely malnourished individuals. Some available studies on other bird species might support this deduction. The studies on Barn swallows (*Hirundo rustica*) and Marsh harriers (*Circus aeruginosus*) showed the inter-sibling immune response difference [72,73]. Another study on the House sparrow (*Passer domesticus*) showed that nestlings that received less food from parents showed a weaker immune response [74]. More studies on the physiological and begging behavior differences among siblings of Asian short-toed larks need to be done in the future.

Similar to our body mass result, some studies have found a reduction in body size due to trophic mismatch-induced malnutrition [8,75]. The ecological consequence of this morphological variation is species-specific. A study on the Blue-footed booby (*Sula nebouxii*) showed that the body mass of nestlings decreased when they suffered trophic mismatch, but there was no significant decrease in longevity and reproductive performance in smaller fledglings [75]. On the contrary, a study on a long-distance migrant species, Red knot (*Calidris canutus*), found that the shorter bills of smaller offspring have consequences at their tropical wintering grounds where shorter-billed individuals have reduced survival rates because their bills couldn’t reach deeply buried Loripes [8]. Therefore, the plasticity of immunity and growth may only be a short-term response of nestlings, which could benefit the short-term survival of nestlings suffering trophic mismatch. Due to the different environmental conditions and life history of different species, the long-term fitness consequence of smaller body size needs species-specific research.

## 5. Conclusions

Our results show that a poor food condition stimulates the release of cytokines and inhibits the secretion of IGF-1, reducing the body mass of nestlings. These results indicate that nestlings have immunity and growth plasticity when food is limited. The fact that more than 60% of nestlings could survive even when NBP was at its lowest indicates that the plasticity of immunity and growth may make most of the nestlings survive in poor food conditions, thus partially buffering the effect of climate change-induced trophic mismatch. Such plasticity could only benefit the short-term survival of nestlings suffering trophic mismatch. At the same time, long-term fitness consequence of smaller body size needs species-specific research at different adult life history stages.

## Figures and Tables

**Table 1:** Primer sequences and anticipated size of amplified products.

Gene	Forward Primer	Size (bp)	Accession No.
*TNF-α*	F:5-CCGCCCAGTTCAGATGAGTT-3 R:5-GCAACAACCAGCTATGCACC-3	130	MF000729.1
*IFN-γ*	F:5-TGAGCCAGATTGTTTCGATG-3 R: 5-CTTGGCCAGGTCCATGATA-3	248	NM_205149.1
*IL-1β*	F:5-ACTGGGCATCAAGGGCTACA-3 R:5-GCTGTCCAGGCGGTAGAAGA-3	142	NM_204524.1
*GAPDH*	F:5-CACTGTCAAGGCTGAGAACG-3 R:5-GATAACACGCTTAGCACCA-3	187	NM_204305.1

**Table 2:** Results of linear mixed models of the effects of nymph biomass proportion (NBP) and nest temperature on the expression of the interferon γ (*IFN-γ*), tumor necrosis factor-α (*TNF-α*), interleukin-1β (*IL-1β*) genes and the plasma insulin-like growth factor-1 (IGF-1) of Asian short-toed lark (*Alaudala cheleensis*) nestlings. NBP is the proportion of the daily grasshopper nymph biomass relative to the total biomass measured on all survey days.

Response Variable	Explanatory Variable	*F*	*p*
*IFN-γ*	NBP	64.015	<0.001
	Nest temperature	1.004	0.323
	NBP × Nest temperature	0.262	0.611
*TNF-α*	NBP	59.585	<0.001
	Nest temperature	3.343	0.075
	NBP × Nest temperature	0.049	0.826
*IL-1β*	NBP	53.372	<0.001
	Nest temperature	3.584	0.066
	NBP × Nest temperature	2.578	0.117
*IGF-1*	NBP	20.675	<0.001
	Nest temperature	2.342	0.134
	NBP × Nest temperature	0.001	0.970

## Data Availability

The data used in the present study are available from the corresponding author upon reasonable request.
